# The roles of vision and proprioception in spatial tuning of sensory attenuation

**DOI:** 10.1007/s00221-024-06982-w

**Published:** 2025-01-11

**Authors:** Clara Fritz, Manuel Bayer, Eckart Zimmermann

**Affiliations:** Institute for Experimental Psychology, Heinrich Heine University Düsseldorf, 40225 Düsseldorf, Germany

**Keywords:** Sensory attenuation, Spatial tuning, Self-touch, Vision, Proprioception, Biological sciences, Psychological and cognitive sciences

## Abstract

When we touch ourselves, the pressure appears weaker compared to when someone else touches us, an effect known as sensory attenuation. Sensory attenuation is spatially tuned and does only occur if the positions of the touching and the touched body-party spatially coincide. Here, we ask about the contribution of visual or proprioceptive signals to determine self-touch. By using a 3D arm model in a virtual reality environment, we dissociated the visual from the proprioceptive arm signal. When a virtual arm was visible indicating self-touch, we found that sensory attenuation generalized across different locations. When no virtual arm was visible, we found sensory attenuation to be strongest when subjects pointed to the position where they felt their arm to be located. We conclude that the spatial tuning of tactile attenuation depends on which signal determines the occurrence of self-touch. When observers can see their hand, the visual signal dominates the proprioceptive determining self-touch in a single visual snapshot. When only the proprioceptive signal is available, the positions of the touching and the touched body-part must be separately estimated and subsequently compared if they overlap in anatomical space.

## Introduction

**Fig. 1:**
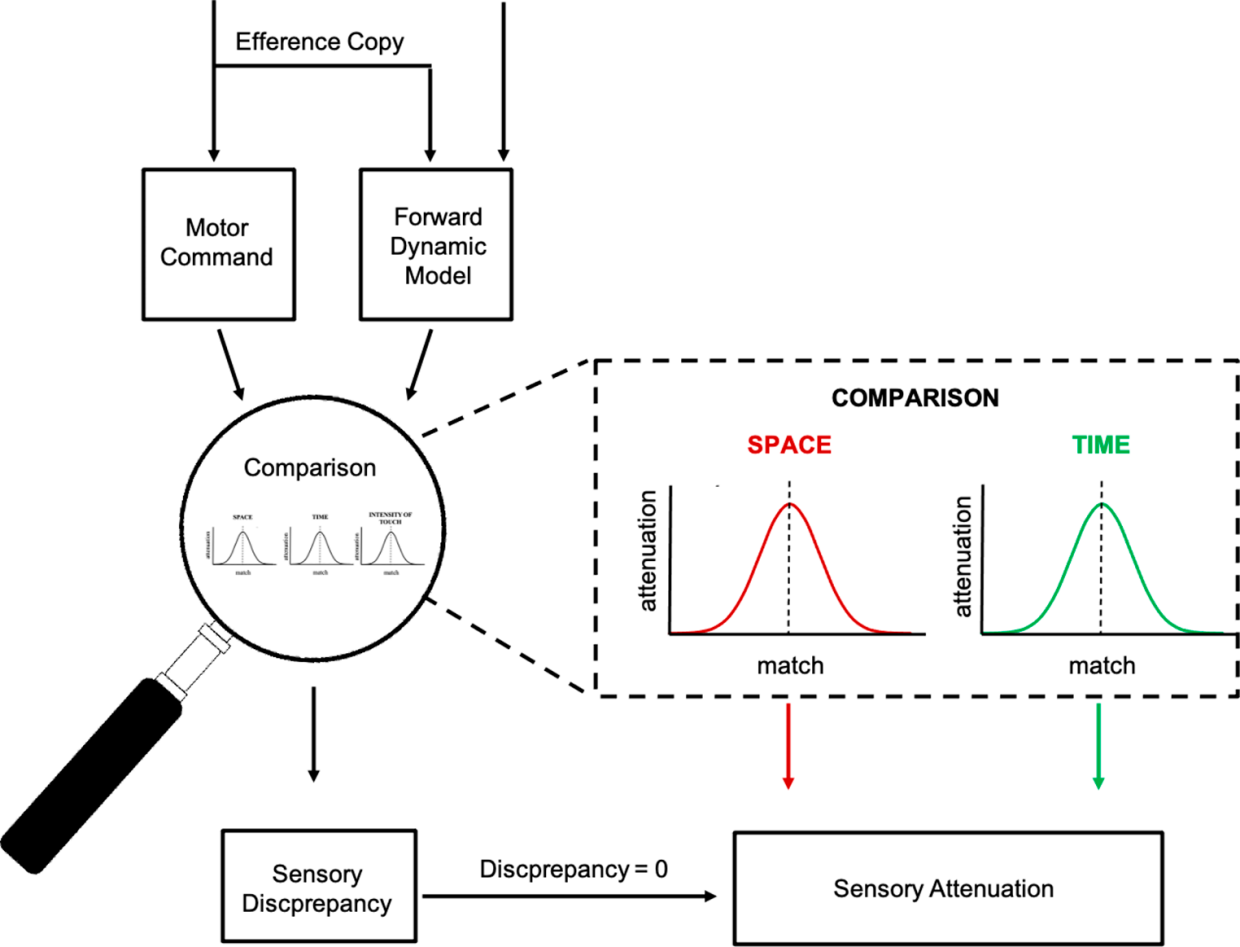
Schematic illustration of the forward model architecture involved in sensory attenuation of self-touch. When the sensorimotor system issues a motor command, an efference copy is generated that contains the information of the upcoming motor vector. The efference copy is used to build up an internal forward model that predicts the sensory consequences of the imminent movement. After termination of the movement, the predicted and the actual sensory consequences of the movement are compared. Where and when the movement results in a touch of a body-part is compared to where and when we feel the tactile sensation. Only if both, space and time match between the estimated and the actual reafferent input signals, sensory attenuation occurs. The distributions show the strength of sensory attenuation in dependence of the spatial and temporal match between the touching and the touched body part. Sensory attenuation magnitude decreases as a function of the anticipation or delay between the felt touch and the applied touch (temporal tuning (Bays et al. 2005; Kilteni et al. 2019). Similarly, sensory attenuation magnitude decreases If the spatial distance between the touched and the touching body part increases (spatial tuning: Bays et al. 2006; Kilteni et al. 2019; Knoetsch and Zimmermann 2021)

When we touch ourselves, we experience the ensuing sensation differently than when someone else touches us (Blakemore et al. 2000; Dogge et al. 2019; Hughes et al. 2013). The most famous example is the inability to tickle ourselves. Apart from the absence of ticklishness experiences, we also feel a self-touch as less intense, a phenomenon termed sensory attenuation (Bays et al. 2006; Blakemore et al. 1999). Sensory attenuation is considered the classic example of how internal predictions about the sensory consequences of our actions shape our perception (Blakemore et al. 1999, 2000). Distinguishing sensations produced by stimuli in the external world from sensations generated by our movements is vital for successful interaction in our environment. For instance, if we were not able to predict that stimulation on the retina is produced by our own eye or head movements, we would constantly feel dizzy and incapable of keeping balance. Predicting the tactile sensations of self-touch might be important to avoid the alarming reaction that we experience when small animals crawl across our skin. If the actual consequences of our actions match the predictions, we experience these sensations as less intense (Blakemore et al. 2000). Brain imaging studies found that in somatosensory cortex processing is attenuated for the predicted consequences of our actions (Hesse et al. 2010). In order to provide a clear-cut distinction between externally and internally generated sensations, predictions must be precise. Deviations of the actual sensations from the predictions should be experienced since these might be caused by external events that we should be aware of. Predictions of the sensory consequences of our movements are provided by the so-called forward model, which is built up by the efference copy (Desmurget and Grafton 2000; Miall and Wolpert 1996). Once the movement is executed, the brain compares the predicted sensory consequences from the forward model with the actual sensory feedback. This comparison allows the brain to detect any sensory discrepancies between the expected and actual outcomes (Von Holst 1954; Wolpert et al. 1998). However, to this end, a fine-tuned process must compare predicted and actual sensations (see Fig. 1).

Studies reported fine temporal and spatial tuning of the sensory attenuation effect (Bays et al. 2005; Blakemore et al. 1998; Hughes et al. 2013; Kilteni et al. 2019; Knoetsch and Zimmermann 2021). If the coincidence between the time of the touch and the felt experience on the other finger was artificially delayed, sensory attenuation was not observed (Bays et al. 2005). Similarly, the spatial distance between the touching and the touched finger determines the strength of sensory attenuation (Bays and Wolpert 2007; Kilteni et al. 2019; Knoetsch and Zimmermann 2021). However, the spatial tuning of sensory attenuation must be evaluated carefully. Does a mechanism calculate the spatial distance between the touching and the touched finger or does vision of both hands simply confirm or disconfirm if self-touch took place? The latter process arguably requires fewer computational resources compared to the former since only a single visual judgment answers the question. The requirements to determine the occurrence of self-touch differ between the visual and the proprioceptive modality. Visual categorization processes can assert self-touch by relying solely on the information present in the retinal image. However, when only input from the proprioceptive sense is given, a spatial comparison is necessary to determine self-touch. In the case of contact between both hands, the spatial position of the left and the right hand have to be compared in order to distinguish if both hands either touched each other or a third object.

Here, we tested how vision and proprioception each contribute to the spatial comparisons of the touched and touching body-part. We tested sensory attenuation of self-touch in a virtual reality (VR) environment in which we presented 3D arm models such that the touching arm was controlled by the participant’s arm movements. Using a VR environment allowed us to manipulate the position of touch in relation to the position where tactile stimulation took place. In separate sessions, the virtual hand was either visible or not. With these two manipulations (spatial distance between the touch and the ensuing tactile sensation and visibility of the visual arm), we sought to determine the contributions of the visual and the proprioceptive sense for the localization of the arm during the prediction of self-touch.

A direct comparison of sensory attenuation magnitude between the hand visible and hand invisible conditions is contaminated by the fact that non-informative vision of the hand enhances tactile discrimination (Cardini et al. 2012; Eads et al. 2015; Harris et al. 2007; Kennett et al. 2001; Taylor-Clarke et al. 2002; Suzuishi and Hidaka 2022). The same holds true also for tactile discrimination sensitivity during reaching to grasp (Colino et al. 2017) and grasping movements (Juravle et al. 2018; Voudouris and Fiehler 2022). Tactile discrimination tasks like those usually applied in sensory attenuation of self-touch tasks will thus yield different results when the hand is seen or not seen. However, to the best of our knowledge, nothing is known about the interaction of tactile discrimination by non-informative vision and sensory attenuation. In the current study, we analyzed the spatial specificity of sensory attenuation separately for stimulation of visible and invisible hands.

## Materials and methods

All experimental procedures were carried out in accordance with the ethical standards of the Declaration of Helsinki from 2024 and were approved by the local ethics committee of the Faculty of Mathematics and Natural Sciences of Heinrich Heine University, Duesseldorf (identification number: 757184). Participants were recruited at the University of Duesseldorf and were compensated with participation hours or remunerated by means of an expense allowance. Informed consent was obtained from all participants. One participant had to be excluded because we were unable to fit the data to a psychometric function (please see data analysis section for more information). For the 59 participants, age ranged from 18 to 32 years, with *M*_*Age*_ = 22.07 ± 3.29 (*SD*), 40 females. 11 had vision correction. Handedness was assessed using the Edinburgh Handedness Inventory and all participants were classified as right-handers (*M* = 90, *SD* = 15.14).

### Baseline measurement

**Fig. 2:**
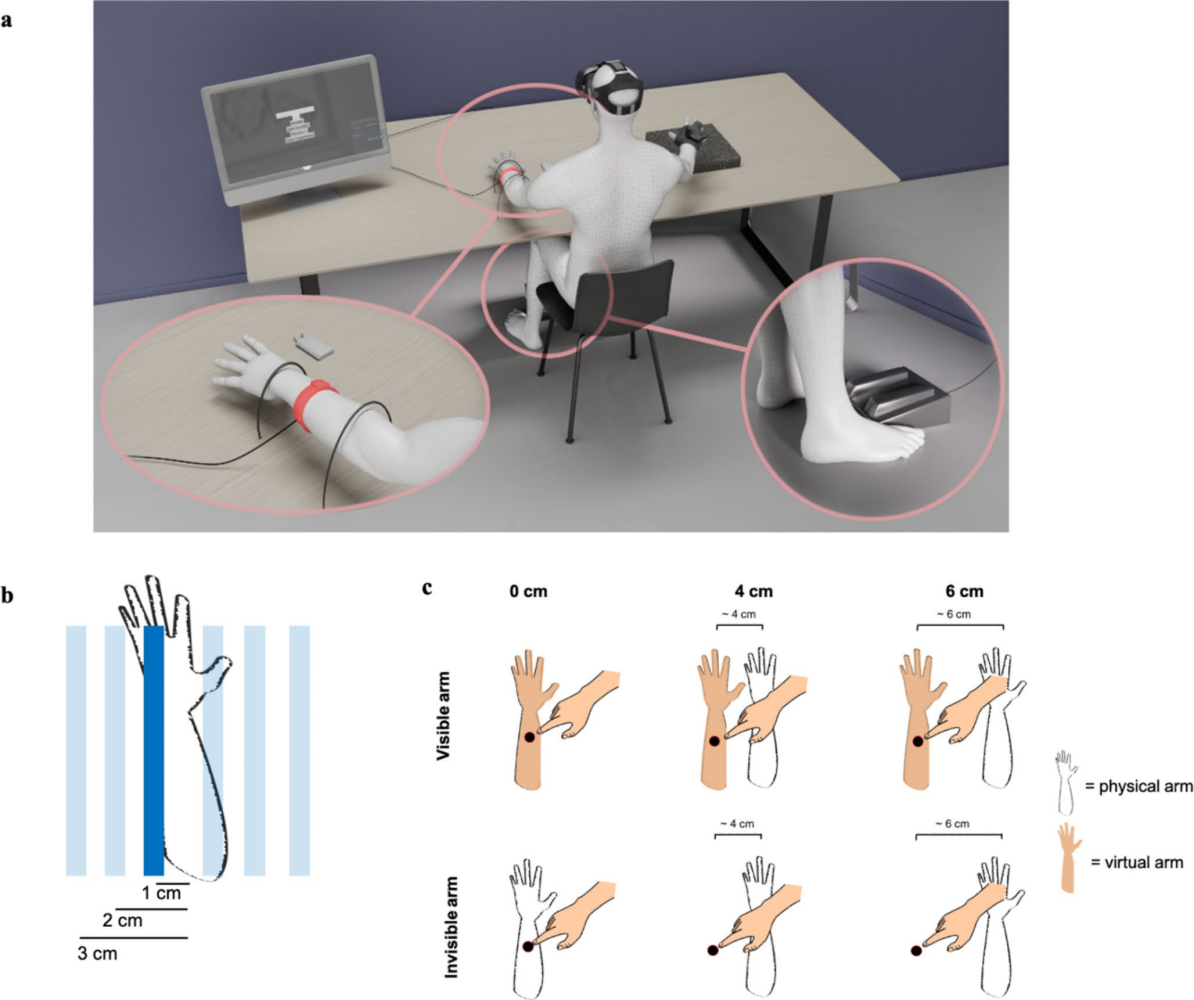
Experimental setup. (**A**) Participants were seated in front of a table and equipped with a VR headset. A vibromotor was attached to their physical left arm, while their physical right hand rested on a tracking pad with a Vive tracker securely attached. A foot pedal underneath the table was used to record the responses of the participants. (**B**) Experimental design of the baseline measurement. Subjects were instructed to compare the position of the visual bars against the midline position of their arm. The midline of the arm would thus correspond to 0 cm. In a trial, one bar (indicated by the dark blue bar) was shown − 3, -2, -1, 1, 2, 3 cm (indicated by the light blue bars) relative to the unseen arm midline position. (**C**) Positions of the physical and virtual arm in Experiments 2 and 3. Participants were required to touch a virtual dot presented either on their physical arm (outlined transparently with a black frame) or on two outer positions 4–6 cm further to the left of their physical arm. Within VR, the left-hand position was either visible (see top row) or invisible (see bottom row)

We first measured the sensitivity to discriminate the location of a visual stimulus against the proprioceptively felt location of the left arm (baseline measurement). Participants were seated in front of a table in a quiet lab environment wearing a VR headset (HTC Vive Pro) and headphones including a noise canceling function (Soundcore Q30) (see Fig. 2A). Experiments were conducted on an Alienware Aurora R13 computer (Windows 10, Intel(R) Core(TM) i7-12700 F, 2.1 GHz, NVIDIA GeForce RTX 3060). The VR setup was implemented within the Steam VR beta (version 1.22.9) software. Using the VR headset, participants were immersed in a virtual environment that closely resembled the physical world. The laboratory, in which the experiment took place, was recreated within VR. The participant’s physical left arm was resting on the table and was fixated with two plastic loops. Participants right hand was placed on a pad 60 cm to the right of participants left arm. A vive tracker was attached to participants right hand so all movements were directly transferred within the virtual environment. Experimental instructions were displayed in red on the black wall in front of participants. All experiments were conducted in the virtual environment and participants saw a virtual replica of the table in front of them in the virtual world. In the baseline measurement, participants were required to judge whether a visual bar stimulus appeared to the left or to the right of their unseen physical arm (see Fig. 2B). The bar was oriented parallel to the arm and in each trial, the bar appeared in one of six possible locations. Response was given with the help of the foot pedal. Participants were asked to enter whether the first or second stimulus was stronger by either pressing the left or the right side of the foot pedal. After entering a response, the next trial started automatically. In a baseline measurement, 60 participants were tested.

### Experiment 1

In Experiment 1, we aimed to replicate the classic sensory attenuation effect for self-touch within our experimental setting. Experiment 1 contained two conditions that were tested in separate sessions: An active reaching and a passive no-movement condition. In the active reaching condition, subjects were seated in front of a table with their physical left and right arms placed in front of them. The physical environment was rebuilt within the virtual environment so that in VR participants were seated in front of a virtual table. Both of their arms were presented as visual 3D arm models. Participants’ left arm was laying on the table, covered by two 3D self-printed plastic loops to prevent participants from changing their arm position. A vibromotor (Adafruit Mini Vibrating Motor Disc Buzzing Motor, 10 mm Diameter), connected to an Arduino nano microcontroller ATmega328 operating at 5 V and through a pulse width modulation module (TS-YM-303), was attached on the top of subjects’ left forearm using velcro. With the help of a custom-made program in Python (version 3.10.2) and Arduino (Version 1.8.15) we were able to output seven different vibration strengths to the vibration motor by operating at 1.44 V to 4.62 V with 0.53 V differences between level.

A trackpad served as an interface bridging the virtual and physical environments. In the physical world, participants were able to rest their hand gently on the trackpad between trials. Simultaneously, in the virtual world, the trackpad enabled us to monitor whether participants’ hands returned to the starting position, as the trackpad remained stationary between trials. This stability allowed us to track hand positioning with precision across tasks, ensuring accurate measurement of hand movement and return to the initial position between trials.

After positioning their right hand on the trackpad, a trial started. In the virtual environment, a blue dot (radius: 0.5 cm) appeared 1.5–2 s after trial start on the subject’s left virtual arm. The subjects were instructed to touch the dot with a reaching motion. To control for correct movement execution, the dot turned green (an indicator that the movement was correct) when the reaching criteria were met. A reaching movement was only considered executed correctly if participants’ right hand was 15 cm above the table during 75% of the trajectory. Only when the movement was executed correctly, stimulations were presented to the left arm. In case the movement was too flat, movements had to be repeated. All trials were included in the analysis as participants were allowed to repeat the movement. Movement onset and offset were tracked with the help of the vive controller attached to the participant’s right hand. The vibration on the left arm was triggered whenever a sphere with a radius of 0.5 cm around the left arm and another sphere around the right index finger (radius: 0.5 cm) overlapped. Participants were instructed to touch their left arm briefly and then move it back to the trackpad. When touching the left-hand participants were able to feel the vibration motor on their right index finger. When touching the dot, a vibration occurred on the corresponding physical arm position through the vibration motor for 300 ms with an intensity of 3.03 V. 750 ms later, a second vibration appeared, varying in its intensity between six equal distance steps from 1.44–2.5 V and 3.56–4.62 V. An invisible sphere with a diameter of 0.75 cm surrounded the finger tip of the touching finger. Another invisible sphere with a diameter of 1 cm surrounded the target location. As soon as both spheres overlapped (checked with the 90 Hz frame rate), the vibration was triggered. The microcontroller has a latency of 5 ms and the USB adapter a latency of approximately 2 ms, together with the 12 ms of one frame adds up to a maximum latency of around 20 ms in total.

The position of the virtual dot matched the midpoint of their physical arm between the wrist and the elbow. When the reaching finger arrived at the position corresponding to that of the virtual dot, a probe vibration occurred on the physical left arm applied by the vibration motor. Subjects had to judge whether the probe vibration appeared weaker or stronger in intensity than a reference vibration which occurred 750 ms later. Participants responded on the foot pedal if the first or second vibration was perceived as stronger. It was randomized between participants whether the first or second vibration referred to the left or right foot pedal. The experimental setup is shown in Fig. 2A. Each stimulus level was presented 10 times and 60 trials were conducted in total. After the experiments, subjects filled out a custom-made questionnaire where they were asked if anything unusual happened throughout the study or whether they felt like touching their own arm. In the passive no-movement condition, the trial structure remained identical, except that participants did not have to perform an active reaching movement. Instead, the probe vibration was delivered automatically 1000 ms after the trial start and 750 ms later the reference vibration. In the passive movement condition, trials started after participants right hand was placed on the track pad. Probe and reference vibrations were presented to participants left arms with the same timing, duration and intensity as before.

In Experiment 1, from originally 53 participants, three data sets were excluded from further analysis since their data did not allow to fit a psychometric function. For the final analysis data of 50 right-handed participants between the age of 18 and 34 (*M*_*Age*_ = 21.98 ± 6.64 (*SD*), 28 females) were analyzed. In Experiment 1, participants underwent one passive no-movement and one active reaching condition. The order of conditions was counterbalanced across participants.

### Experiment 2

In Experiment 2, we aimed to spatially dissociate the seen and the proprioceptively felt arm position in an active reaching and a passive no-movement condition. We mounted two further vibromotors on top of a custom-made plastic surface, functioning as a fake arm object (in the following referred to as the shifted 4 cm position for the vibromotor position right next to the left arm and the shifted 6 cm position for the furthest vibromotor on the left side). When touching the small vibromotor all three surfaces of the various positions felt identical. Due to the varying width of the subjects’ forearms, we had a mean distance between the middle of the subject’s left arm and the close fake arm of *d* = 4.1 and between the middle of the subjects’ left arm and the far fake arm of *d* = 6.1.

To start a new trial, participants were asked to fixate a blue cross in the upper right corner of their field of view for 500 ms. Next, a blue dot appeared on the left side of their field of view, localized on their left visual arm. Physically, the virtual dot matched either the midpoint of the physical arm, or a position physically shifted 4–6 cm.

During the active reaching condition participants task was to touch the virtual dot. When touching the green dot, participants felt probe and reference vibrations on their physical arm with the same timing duration and intensities as before. Participants responded by either pressing the left foot pedal if the first stimulus felt stronger and the right foot pedal if the second stimulus felt stronger or vice versa. The order of pedal mapping was randomized between participants. Participants completed two sessions with 90 trials each. During the sessions, the trials were presented randomly on the subjects’ physical arm, the 4 cm shifted position, or the 6 cm shifted position, with 10 trials for each vibration strength for each position, resulting in 180 trials in total.

After each trial, participants were asked to fixate on a blue cross in their top right visual field of view. Following the fixation, a black screen was flashed for 500 ms, during which the position of the visual left arm was shifted. During fixation, the left arm was outside the participants’ field of view, so the visual arm shift took place unobtrusively. As we varied the visual arm position between trials, participants always had the visual feedback of touching their virtual arm. However, physically, in two third of all trials, they touched either the 4 cm or the 6 cm shifted fake object. At the end of the experiment, participants were asked in a questionnaire if they noticed the arm shift. Based on their responses, three participants were excluded from the analysis. Participants completed both, the passive no-movement condition and the active reaching condition.

For Experiment 2 (including visible passive no-movement and visible reaching condition), we included data from *N* = 57 participants. Three participants had to be excluded from the originally 60 participants as they were able to describe the visual manipulation. Age ranged from 18 to 37 years, with *M*_*Age*_ = 23.17 ± 4.31 (*SD*), 42 females. 15 had vision correction and all were right-handed.

### Experiment 3

In Experiment 3, we aimed to dissociate the physical arm position from the perceived arm position of participants. The procedure was identical to Experiment 2. However, in Experiment 3, the virtual left arm was not visible in virtual reality (VR). Similar to Experiments 1 and 2, we conducted an active reaching and a passive no-movement condition. In the active reaching condition, the position of the virtual dot either matched participants’ physical arm or one of two fake objects in the physical world (4 and 6 cm shifted to the left of the physical arm). To start a new trial in the active condition, participants were asked to place their physical right hand on the trackpad in front of them and fixate a blue cross in the upper right corner of their field of view for 500 ms. Next, a blue dot appeared on the left side of their field of view, localized either on their arm, the shifted 4 cm position in the physical world, or the shifted 6 cm position in the physical world. In all three scenarios participants felt a vibration motor when touching with their right index finger. The left virtual arm was invisible and targets were only presented at the same height as the upper part of the left arm. The height was measured in a calibration phase at the beginning of the experiment. Subjects were asked to perform the same active reaching trials as described in Experiments 1 and 2. When participants touched the position that was indicated by the blue dot, a probe vibration occurred, followed by a reference vibration 750 ms later. As in Experiments 1 and 2, the task of the subjects was to judge whether the first or second tactile impulse was more intense.

We also measured a passive no-movement condition in this experiment, as described in the section *Stimuli and tasks for Experiments 1–3*. Participants were seated and asked to judge the intensity of two vibrations presented to their left arm. The virtual left arm was invisible in this condition.

In Experiment 3 we tested *N* = 70 participants in the invisible reaching condition. Their age range varied between 16 and 52 (*M*_*Age*_ = 22.09 ± 7.8 (*SD*), 28 females). 18 participants had vision correction in the form of glasses or contact lenses, and all were right-handed. In the invisible passive no-movement condition, we analysed data of *N* = 48 participants (age range: 19 to 36, *M*_*Age*_ = 24.01 ± 5.5 (*SD*), 32 females, 9 with vision correction).

### Data analysis

Data preprocessing was performed in R (Version 4.0.3). We first calculated mean correct responses per stimulus level for each subject. We then fitted these data with cumulative Gaussian functions to determine psychometric functions. The mean of the psychometric functions estimated the Point of Subjective Equality (PSE). We chose the standard deviation as the measure of the just noticeable difference (JND). Statistical analysis was performed in JASP 0.16.3 (Intel). To compare average results statistically, we used repeated measures ANOVAs and dependent t-tests.

## Results

### Spatial estimates between vision and proprioception are precise – baseline measurement

In the baseline experiment the sensitivity to discriminate the location of a visual stimulus against the proprioceptively felt arm location was measured. We estimated psychometric functions with a least square fit and extracted the Point of Subjective Equality (PSE) and the Just Noticeable Difference (JND) for every participant. The average PSE across all participants was *M* = 0.05 cm ± 1.48 (*SD*), showing that subjects’ bias was close to zero, which would equal perfect localization accuracy. The JND of *M* = 1.17 cm ± 0.55 (*SD*) indicates a precision of 1.17 cm in relating proprioceptive space to visual space. In other words, only within 1.17 cm around the midpoint of their arm, subjects were uncertain when relating the position of a visual stimulus to their arm.

### Sensory attenuation for self-touch is replicable in VR – Experiment 1

**Fig. 3:**
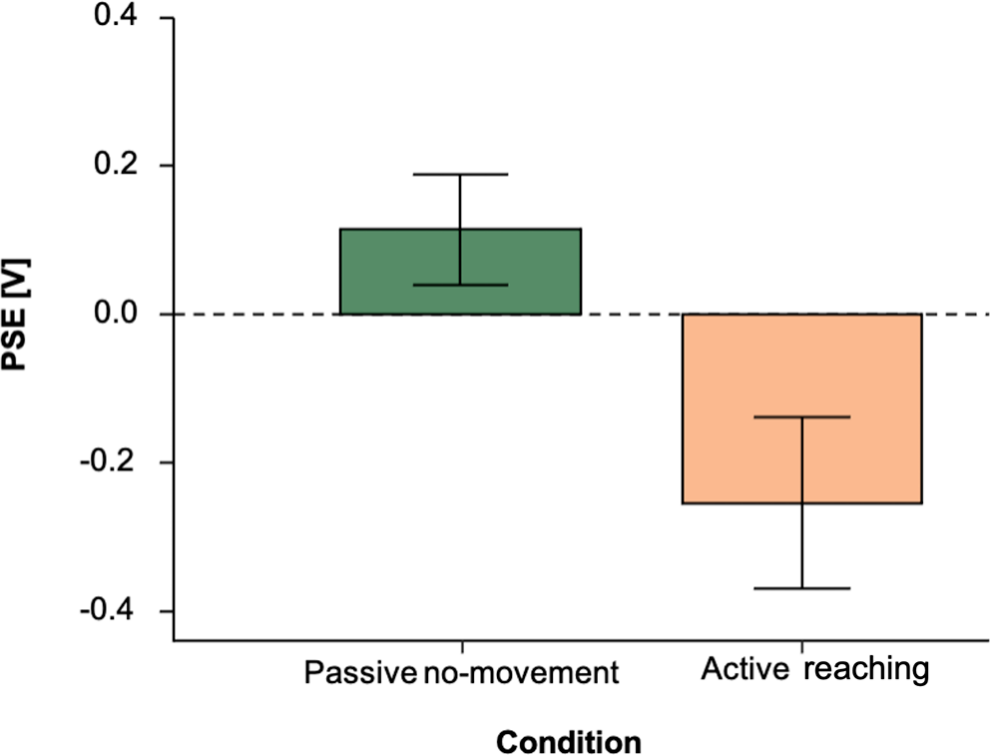
Barplots for results of Experiment 1. Mean perceived stimulus strengths are shown for the passive no-movement and the active reaching condition. Group conditions are shown against the PSE. Error bars represent S.E.M. Dashed grey line represents physical equality between probe and reference stimulus

Participants underwent one passive no-movement and one active reaching condition. For both conditions, we fitted individual psychometric functions for every participant. Mean PSE values for the two conditions are shown in Fig. 3. The green bar represents the passive no-movement condition, whereas the beige bar represents the active reaching condition. In the passive no-movement condition, we found a mean PSE value across participants of *M* = 0.12 [V] ± 0.54 (*SD*). Thus, a mean of 0.12 V represents a slight overestimation of the reference stimulus. In the passive no-movement condition, intensity values only slightly deviated from 0. The corresponding average JND of 0.65 [V], with a range of ± 0.39 (*SD*), suggests a precise discrimination ability in this condition. Comparably, in the reaching condition, participants underestimated the intensity of the probe stimulus with *M*_*PSE*_ = -0.22 [V] ± 0.78 (*SD*). We found a significant difference between the PSEs of the two conditions (paired t-test, *t*(49) = -2.35, *p* =.023, *d* = -0.332, CI [-0.62, -0.05]), replicating the classic effect of sensory attenuation. Probe vibrations in the reaching condition, including self-touch, were significantly attenuated compared to the passive no-movement condition. The discrimination sensitivity in the reaching condition was *M*_*JND*_ = 0.72 [V] ± 0.46 (*SD*). Sensitivity was statistically indistinguishable between conditions (t-test, (*t*(49) = 0.78, *p* =.439, CI[-0.17, 0.39])).

### Visual observation for self-touch generalizes across space – Experiment 2

**Fig. 4:**
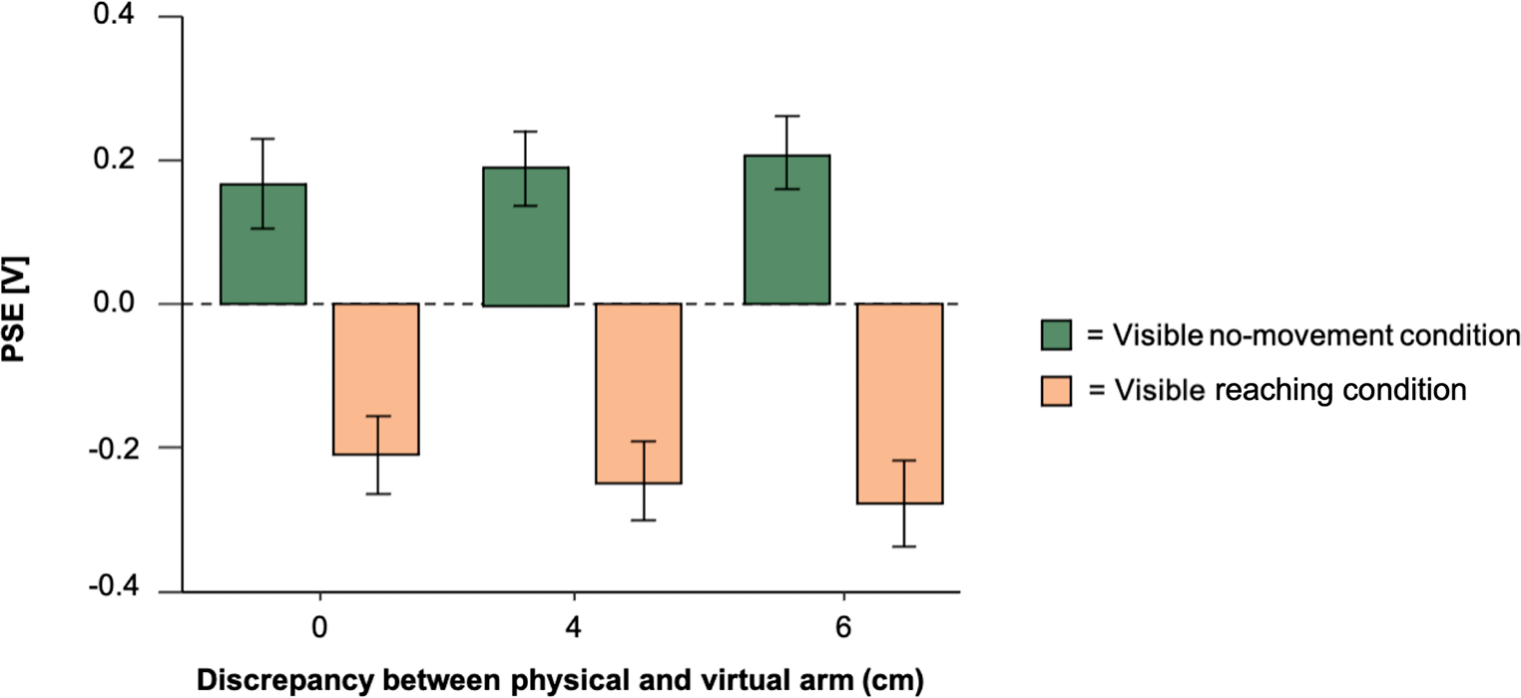
Results of Experiment 2. The PSE indicating the perceived tactile intensity is shown as a function of the discrepancy between the physical and the virtual arm for the conditions of visible passive no-movement and visible active reaching. Error bars represent S.E.M. Dashed grey line represents physical equality between probe and reference stimulus

In Experiment 2, the seen and the proprioceptively felt arm position where spatially dissociated in an active reaching and a passive no-movement condition. Participants underwent 60 trials for each of the three arm positions. We calculated psychometric functions for all arm positions, for each condition and each participant. PSEs barely changed between the different arm positions (for the physical arm with *M* = -0.21 [V] ± 0.63 (*SD*), 4 cm position with *M* = -0.25 [V] ± 0.63 (*SD*) and 6 cm position with *M* = -0.277 [V] ± 0.63 (*SD*)). The PSEs of the reaching condition are displayed with beige bar plots in Fig. 4. The passive no-movement condition was conducted similarly to the passive condition in Experiment 1. The virtual left arm was visible within the virtual environment, and we presented the visible arm at three positions, matching the physical arm, or the 4 and 6 cm fake object positions from the physical world. The virtual left arm was displaced inconspicuously for participants between trials. As the same subjects in the reaching to a visible arm experiment participated in both, the passive no-movement and the active reaching condition, we conducted a 2 × 3 repeated measures ANOVA with the factors condition (no-movement vs. reaching) and discrepancy between physical and virtual arm (0, 4, 6 cm). We found no significant effects for the factor position (*F*(2, 112) = 0.034, *p* =.966) and the interaction (*F*(2, 112) = 0.475, *p* =.623). We found a significant effect for the factor condition (*F*(2, 112) = 29.072, *p* <.001, η² = 0.185), suggesting that attenuation effects were significantly stronger in the reaching condition compared to the passive condition as the probe stimulus was perceived attenuated. Figure 4 shows the discrepancy in attenuation between the two conditions. The same analysis (2 × 3 repeated measures ANOVA) for the JND values did not reveal any significant effects for the factor condition (*F*(1,56) = 0.138, *p* =.712), for the factor position (*F*(2,112) = 0.228, *p* =.797) nor for the interaction effect (*F*(2,112) = 0.673, *p* =.512).

### Sensory attenuation is spatially tuned in proprioception – Experiment 3

**Fig. 5:**
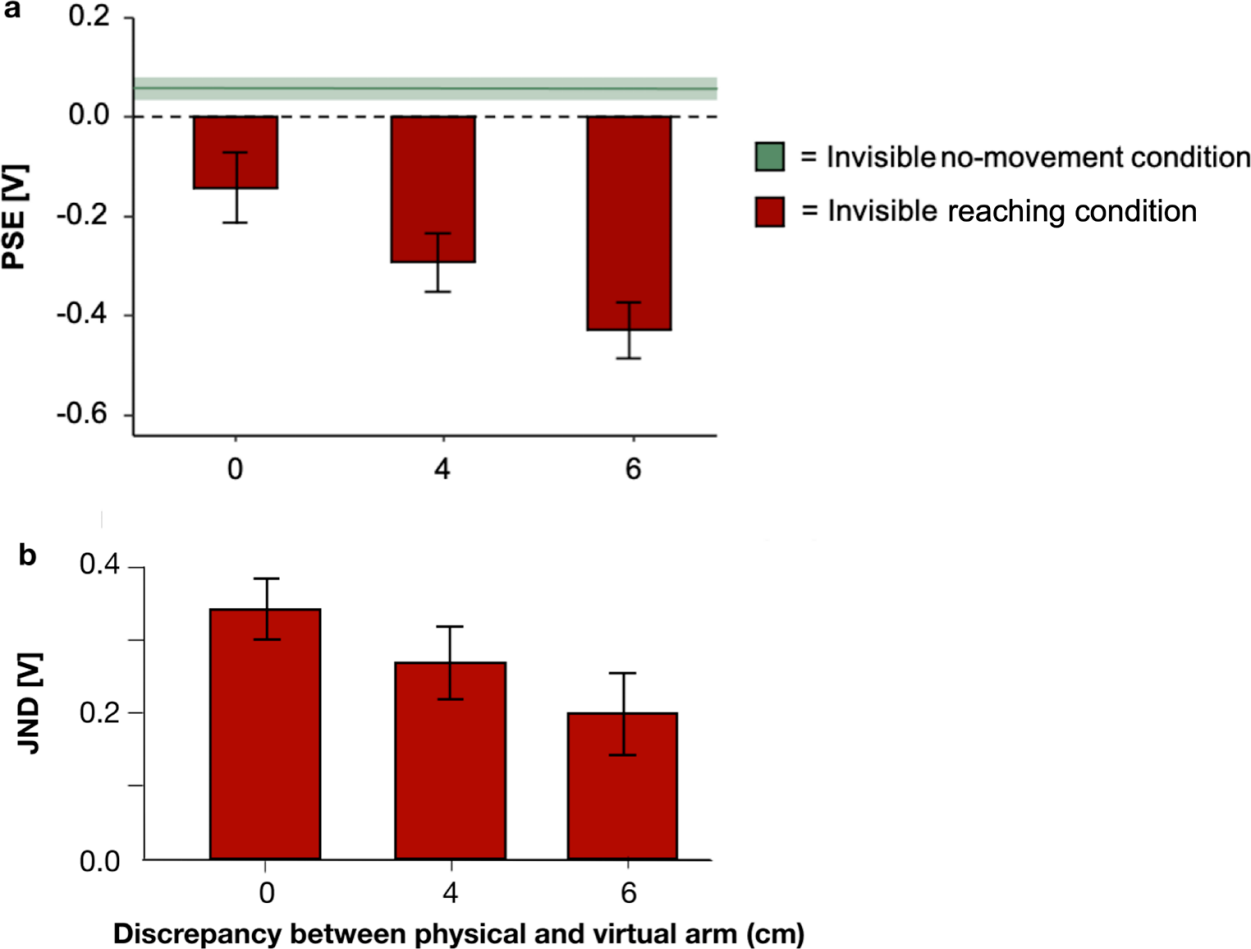
Results of Experiment 3. (**a**) Average PSEs indicating the perceived tactile intensity are shown against the discrepancy between the physical and the virtual arm. Positive numbers represent an overestimation of tactile intensity of the first stimulus. Error bars represent S.E.M. Dashed grey line represents physical equality between probe and reference stimulus. (**b**) Average JNDs shown against the discrepancy between the physical and the virtual arm. Error bars represent S.E.M

Experiment 3 was identical to Experiment 2 except that during the passive no-movement and active reaching condition, the virtual left arm was invisible in VR. We calculated PSE and JND values for judgments of tactile impulses for each arm position and for each participant. Negative PSE values indicate stronger attenuation. It became evident that attenuation increased when the reaching position was extended further outward. Specifically, the PSE on the physical arm was − 0.14 [V] ± 0.77 (*SD*), whereas the shifted 4 cm position led to a PSE of -0.29 [V] ± 0.66 (*SD*) and the shifted 6 cm position to a PSE of -0.43 [V] ± 0.61 (*SD*). This indicates an increase of attenuation as a function of the eccentricity of the reaching position (one-way repeated measures ANOVA *F*(1.882, 129.837) = 5.299, *p* =.007, due to violation of sphericity Huynh-Feldt results are reported). Barplots showing the mean PSEs of the three conditions can be seen in Fig. 5. The x-axis includes the three arm positions. Attenuation for the probe stimulus was strongest at the shifted 6 cm position. Post-hoc tests reveal a significant effect between the real arm and the 6 cm position (Bonferroni-corrected *p*/3 = 0.004). The JNDs between the three arm positions did not vary. A one-way repeated measures ANOVA did not reveal a significant effect. Again, as sphericity was violated in the dataset, we report Huynh-Feldt results: *F*(1.841, 127.057) = 2.114, *p* =.129.

A second sample of participants performed a passive no-movement condition. The subjects felt two tactile vibrations and were asked to judge which tactile impulse was perceived as stronger. The averaged PSE across participants was *M* = 0.07 [V] ± 0.622 (*SD*). We conducted independent samples t-tests between the PSE of the passive no-movement condition and individual PSEs of all three arm positions to find out whether sensory attenuation was present for all positions. We report Bonferroni-corrected p-values for multiple comparisons. We found significant effects between the passive no-movement condition and the 4 cm shifted position (*t*(116) = 2.965, *p* =.004, *d* = 0.556, CI [-0.93, -0.18]) as well as the passive no-movement condition and the 6 cm shifted position (*t*(116) = 4.293, *p* <.001, *d* = 0.804, CI [0.42, 1.18]), but not between the passive no-movement condition and the physical arm (*t*(116) = 1.564, *p* =.121, CI [-0.78, 0.66]).

The same analysis with JND values revealed significant differences between the passive baseline and the real arm (*t*(116) = -3.615, *p* <.001, *d* = -0.667, CI [-1.05, -0.18]) as well as the passive no-movement condition and the 4 cm shifted position (*t*(116) = -2.42, *p* =.017, *d* = -0.45, CI [-0.82, -0.08]), and 6 cm shifted position (*t*(116) = -2.55, *p* =.012, CI [-0.85, -0.10]). The analysis thus reveals that reaching to invisible targets impedes the discrimination of tactile stimuli.

### Visual observation of self-touch overrides proprioceptive spatial tuning

**Fig. 6:**
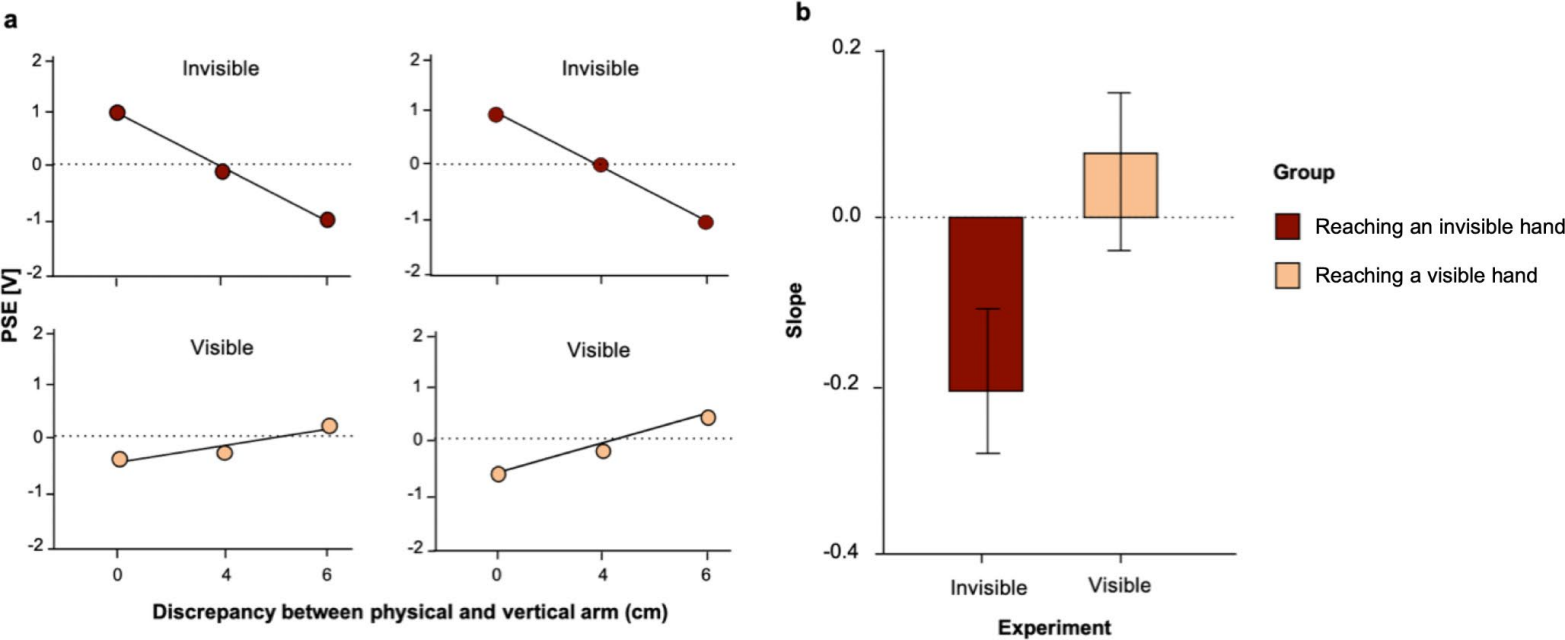
Comparison between active reaching to a visible (Experiment 2) and an invisible arm (Experiment 3). (**a**) Example slopes for two example participants in the invisible and visible experiment. Color code of conditions is congruent to Figs. 4 and 5. (**b**) Means of the linear regression slope to an invisible or visible hand. Error bars represent the standard errors

In order to directly compare the putative dependence of sensory attenuation on the spatial position, we determined the relationship between intensity PSEs and visuo-proprioceptive hand discrepancy. We fitted a linear regression through the individual PSEs values of each participant for the three reaching positions. In Fig. 6A linear regressions for two example participants are shown. For sessions in which they had to point to their invisible hand, the relationship between attenuation and hand discrepancy was negative, implying stronger attenuation as a function of hand discrepancy. By contrast, in sessions in which they had to point to their visible hand, the relationship was positive for these two participants.

We extracted slope and intercept values from the linear regression for each participant. Average slopes from reaching to the invisible hand (shown in red) and from reaching to the visible hand (shown in beige) can be seen in Fig. 6B. We found a significant effect between the slopes of invisible (*M* = -0.206 [V] ± 0.720 (*SD*)) and visible reaching (*M* = 0.058 [V] ± 0.653 (*SD*)): unpaired t-test (*t*(125) = -2.142, *p* =.034, *d* = − 0.382). Intercept values did not differ significantly (*t*(125) = -1.316, *p* =.191).

## Discussion

We investigated how vision and proprioception contribute to the spatial tuning of sensory attenuation. Using VR, we could dissociate the position of the virtual hand seen in the head-mounted display from the physical hand touching the own other hand. Subjects either touched their own hand or they touched a fake object separated by a few centimeters from their physical hand. In the visible condition (Experiment 2), the visual right hand was presented such that the visual right hand always touched the visual left. In the invisible condition (Experiment 3), no hand was visible. In Experiment 2, we found that sensory attenuation magnitude remained unmodulated by the separation between the touching and the touched location as long as visually they seemed to touch themselves. This finding demonstrates that if subjects see both the touched and the touching arm, the comparison process that drives sensory attenuation relies mostly on a visual check determining whether the finger actually touches the arm. This interpretation is in line with the visual dominance in multisensory tasks which involve visual and proprioceptive inputs (Maravita et al. 2003).

In other words, if an arm is visible, the comparison process does not need to estimate the proprioceptive position of the arm since seeing the arm being touched provides sufficient evidence to assume self-touch. Our data show that the visual confirmation of self-touch overrides the need to invoke an extra computation concerning the spatial touch location.

In Experiment 3, subjects were required to point to a visual target. When they reached the target, a vibration was delivered to their right arm. In one third of the trials, the visual target was placed at the position of the unseen right arm. In two thirds of trials, the visual target was placed further outward than the arm. These trials created a discrepancy between the seen and the felt position of the touch. We found spatial tuning for sensory attenuation, i.e., the felt intensity of the vibrations differed significantly depending on the distance between the visual target and the vibration. Sensory attenuation was strongest on the most outward location of the visual target where the distance between the visual target and the vibration was largest. Previous studies have shown that sensory attenuation is strongest when the position of the touching and the touched body part match (Kilteni and Ehrsson 2017; Knoetsch and Zimmermann 2021). A likely explanation for our results is that the proprioceptively felt position of the left arm drifted outward.

The change in proprioception could be caused by the discrepancy between the visual target and the position of the vibration which predominated two-thirds of all trials. It is long known that vision dominates proprioception if their spatial information disagrees (Hay et al., 1965). In the rubber hand illusion for instance, the spatial disagreement between the location of the rubber hand and the tactile stimulation on the real hand is resolved by a spatial drift in proprioception of the real toward the rubber hand (Makin et al., 2008). In our invisible condition there was no virtual hand presented but a visual target to which subjects had to point. The non-visual self-touch rubber hand paradigm is an example of the illusory perception of self-touch induced by the simultaneous execution of a guided action with one hand and the reception of tactile stimulation at the other hand (Aimola Davies et al. 2013). If both hands are placed within a range of 15 cm, subjects report experiencing self-touch and significant proprioceptive drift can be observed. Our paradigm of Experiment 3 is very similar to the non-visual self-touch rubber hand paradigm. Subjects learn that a touch at the visual target elicits a vibration on the physical arm. The maximal distance between both is 6 cm, far below the 15 cm range. Given these similarities, it is likely that our paradigm evoked proprioceptive drift of the physical left arm toward the visual target which was more outward than the arm in two-thirds of all trials. The drift should remain consistent across trials, since in the majority of all trials, subjects point more outward.

The roles of proprioception and vision in sensory attenuation have been investigated before. Kilteni et al. (2017) used the rubber hand illusion to dissociate the visual from the proprioceptive hand location. By moving their right index finger, subjects could control the index finger of the rubber hand. After successfully inducing the rubber hand illusion, the authors measured sensory attenuation under this condition. They found attenuation only when the rubber hand illusion was induced and when the rubber hand was placed in a physiologically plausible position. Their results are in line with our findings. In their setup, vision can determine self-touch in a single glance, and if the rubber hand is not seen touching the other hand, no attenuation occurs. Conversely, if the rubber hand touches the finger, although the real hand does not, sensory attenuation ensues. We conducted our experiments in a VR environment. While we made efforts to maintain a high level of presence and immersion, the artificial nature of the virtual environment may introduce differences in perceptual processing compared to real-world settings. Since it has been demonstrated that even anatomically implausible hands in VR are still perceived as belonging to one’s own body (Yizhar et al. 2021), we did not custom-design the virtual arm for every participant (see supplementary material). Moreover, previous studies showed that small spatial incongruencies do not influence the effects of the Rubber Hand Illusion (Lloyd 2007).

Usually in experiments on self-touch, pressure stimuli are applied (Blakemore et al. 1998; Kilteni et al. 2019). However, in our VR setup, we could only implement vibration stimuli, which are also used to probe tactile sensitivity at the time of arm movements (Colino et al. 2017; Fraser and Fiehler 2018). Since subjects, when they had to touch their real arm (0 cm distance), lightly touched the vibration device, they might have produced a pressure stimulus which subjects felt in addition to the vibration. This additional stimulus would be absent in conditions in which they touched the fake object (4–6 cm distance). The potentially unbalanced stimulation might be a confounding factor in our setup. However, subjects were instructed to lightly tap the position of the arm. The experimenter checked whether subjects performed the touch properly. Although we cannot exclude that subjects felt the touch in addition to the vibration, we judge it unlikely. If the different stimulation would systematically affect our data, it should manifest in Experiments 2 and 3. It should produce differences between the conditions in which subjects touch their real arm and the conditions in which they touch the fake object. However, in Experiment 2 we found very similar sensory attenuation magnitudes for all three conditions.

We conclude that both vision and proprioception contribute to the estimate of self-touch. If both senses are available, vision dominates proprioception. When subjects could see self-touch, no spatial tuning of sensory attenuation was observed. Since vision can confirm self-touch in a single glance, wasting computational resources will be avoided, since in healthy real-life perception, the visual signal representing a body part is never dissociated from its proprioceptive signal. If the visual signal is unavailable, proprioception delivers an estimate of the respective body part position, which is compared to the efference copy signal, representing the position of the reaching finger. Only when both signals match in space, sensory attenuation ensues. In lighted conditions, anatomical and external reference frames are integrated for tactile localization (Badde et al. 2015, 2016; Heed et al. 2024). However, when only proprioception is available, the comparison of the two signals should for economic reasons involve anatomical coordinates only, thus saving the resources for remapping between anatomical and external space coordinates.

### Limitations

We conducted our experiments in a VR environment. While we made efforts to maintain a high level of presence and immersion, it remains uncertain how strong participants accepted the virtual hands as their own. The artificial nature of the virtual environment may introduce differences in perceptual processing compared to real-world settings. For instance, subjects might have accepted larger spatial discrepancies between visual and tactile stimuli because they knew that the artificial environment might not be perfectly aligned. In addition, a previous study has shown that higher cognitive demands in VR can affect tactile sensitivity (McManus et al. 2023). Another limitation concerns the lack of movement tracking data. It has been shown previously that participants move more slowly when they do not see their hand (e.g. Voudouris and Fiehler 2022). However, a critical movement speed is necessary to observe tactile gating (Cybulska-Klosowicz et al. 2011). Although movement behavior was monitored by the experimenter, we cannot exclude that in some trials subjects might have moved too slow for gating to occur.

## Data Availability

Data and code to reproduce analyses have been deposited in OSF available at https://osf.io/k9nuz/.
